# Co-Registration of UAV and Handheld LiDAR Data for Fine Phenotyping of Rubber Plantations with Complex Canopies

**DOI:** 10.3390/plants15030376

**Published:** 2026-01-26

**Authors:** Junxiang Tan, Hao Chen, Kaihui Zhang, Hao Yang, Xiongjie Wang, Ronghao Yang, Guyue Hu, Shaoda Li, Jianfei Liu, Xiangjun Wang

**Affiliations:** 1 College of Earth and Planetary Sciences, Chengdu University of Technology, Chengdu 610059, China; tanjunxiang18@cdut.edu.cn (J.T.); zhangkaihui@stu.cdut.edu.cn (K.Z.); yhao@stu.cdut.edu.cn (H.Y.); wangxiongjie@stu.cdut.edu.cn (X.W.); yangronghao@cdut.edu.cn (R.Y.); hugy@cdut.edu.cn (G.H.); lisd@cdut.edu.cn (S.L.); 2 Rubber Research Institute, Chinese Academy of Tropical Agricultural Sciences, Haikou 571101, China; 3 Chengdu Alundar Technology Co., Ltd., Chengdu 610050, China; jfliu@a-lidar.com

**Keywords:** rubber trees, point cloud, fusion, unmanned aerial vehicle laser scanning, handheld laser scanning

## Abstract

Rubber tree phenotyping is transitioning from labor-intensive manual techniques toward high-throughput intelligent sensing platforms. However, the advancement of high-throughput phenotyping remains hindered by complex canopy architectures and pronounced seasonal morphological variations. To address these challenges, this paper introduces a unified phenotyping framework that leverages a novel Wood Salient Keypoint (WSK)-based registration algorithm to achieve seamless data fusion from unmanned aerial vehicle laser scanning (ULS) and handheld laser scanning (HLS) systems. The proposed approach begins by extracting stable wooden structures through a region-of-interest (ROI) segmentation process. Repeatable WSKs are then generated using a newly proposed wood structure significance (WSS) score, which quantifies and identifies salient regions across multi-view data. For transformation estimation, descriptor matching, WSS constraints, and geometric consistency optimization are integrated into a fast global registration (FGR) pipeline. Extensive evaluation across 25 plots covering 5 sites at the National rubber plantation base in Danzhou, Hainan, China, demonstrates that the method achieves a mean co-registration accuracy of 9 cm. Further analysis under varying seasonal canopy complexities confirms its robustness and critical role in enabling high-precision rubber tree phenotyping.

## 1. Introduction

Phenotypic research in rubber trees provides critical technical support for precision identification and breeding of elite germplasm combining high yield, superior quality, and enhanced stress resistance [1,2,3]. Through systematic characterization of the genetic architecture governing morphological, physiological, and yield-related traits, as well as their environmental response mechanisms, this research substantially accelerates molecular breeding pipelines and optimizes cultivation management strategies [4]. Such advances are pivotal for securing a stable supply of natural rubber—a critical strategic resource—and fostering sustainable development of the industry.

Traditional phenotyping of rubber trees relies on manual sampling and observation, which is time-consuming and labor-intensive, with data often recorded in unstructured forms, hindering systematic quantification, 3D reconstruction, and high-throughput processing, leading to low data utilization, poor traceability, and insufficient standardization [5]. In recent years, advancements in airborne, terrestrial, and portable LiDAR remote sensing have introduced a new paradigm for high-throughput phenotyping [6,7,8]. Among these, the synergistic operation of Unmanned Aerial Vehicle LiDAR (ULS) and Handheld LiDAR (HLS) demonstrates significant advantages in cost-effectiveness, point cloud density, and operational flexibility. Compared to traditional high-altitude airborne LiDAR, low-altitude ULS systems can acquire point cloud data that is at least an order of magnitude denser, offering a cost-effective solution with higher accuracy, thereby enabling refined characterization of the three-dimensional canopy structure. In contrast, HLS systems excel at the real-time 3D reconstruction of the understory, the operational efficiency more than ten times greater than that of traditional terrestrial LiDAR. The integrated ULS-HLS strategy effectively overcomes the technical limitation of traditional methods in achieving both detailed measurement and high operational efficiency within complex canopy structures [9,10], while enabling high-precision 3D spatial data acquisition, thus laying the foundation for automated and standardized phenotypic parameter extraction with structured data.

In rubber plantation scenes, the full realization of this technological potential is ultimately contingent upon the capability to precisely align of multi-source point cloud data, acquired from disparate platforms and viewing angles, into a unified coordinate system [11,12,13]. Global Navigation Satellite System (GNSS) signals are frequently rendered unstable owing to canopy occlusion, resulting in limited accuracy of georeferenced registration approaches [14]. Although artificial targets provide high-precision registration references, their deployment has been associated with substantial costs and practical difficulties in covering extensive forest areas [12,15]. Consequently, marker-free registration methods based on the intrinsic features of point clouds have been extensively utilized.

Feature-based registration methods primarily focus on extracting stable feature primitives and constructing discriminative descriptors to establish reliable correspondences for registration [16,17,18]. Forest scenes are characterized by a lack of structured features, such as lines and planes commonly found in urban environments. Consequently, point primitive matching, which exhibits lower dependency on geometry, has become the mainstream approach. However, traditional keypoint detection methods (such as Intrinsic Shape Signatures (ISSs) [19], Scale-Invariant Feature Transform (SIFT) [20], 3D Harris Corner Detector (Harris3D) [21], etc.) were primarily designed for structured settings, thus exhibiting limited adaptability to the complex canopies.

Researchers are dedicated to developing point cloud registration algorithms for multi-platform forest point cloud registration frameworks [8,9,12,22]. In such frameworks, attributes of individual trees, such as tree height, diameter at breast height (DBH), and stem spatial relationships, are commonly utilized as features to achieve the registration of multi-source point clouds. However, these methods often encounter significant challenges in rubber plantation scenes. This is primarily attributable to the uniform genetic background, monocultural stand structure, and regular planting pattern, which collectively lead to high homogeneity in both tree attributes and spatial relationships, it becomes difficult to construct effective discriminative features for registration [23]. Furthermore, such approaches typically involve computationally intensive preprocessing steps, which further limits their practical applicability [12].

Registration methods have been implemented using either ground or canopy features. Those relying on topographic features [24] have shown limited effectiveness in rubber plantations, which are generally characterized by flat terrain. In contrast, several canopy-based methods have been proposed, including approaches that utilize canopy projection contours [25] or canopy surface fluctuations [14]. However, in rubber plantations with high canopy closure and structural homogeneity, reliably extracting distinctive features remains challenging, resulting in significant degradation of registration performance. While Zhang et al. [22] used uniformly downsampled points as keypoints for direct registration, their even distribution fails to prioritize distinctive areas, thus compromising the matching accuracy across views. To improve matching stability, some studies [13] integrate semantic information to construct keypoints, enhancing the representation of significant areas and keypoint correspondence. However, due to the lack of a stable region segmentation mechanism, keypoints may still fall into unstable regions such as leaves, which are easily disrupted by wind, thus affecting matching robustness in wind-affected forest environments.

In recent years, deep learning has made significant progress in the field of point cloud processing as a powerful tool for automated feature extraction [26,27,28]. Deep learning models, such as fully convolutional networks [29], PointNet [30], and Transformers [31], have provided new approaches for point cloud registration through their strong feature learning capabilities, leading to representative works such as Fully Convolutional Geometric Features (FCGF) [32] and SpinNet [33]. However, applying these models to forest point cloud registration still faces significant challenges: first, model performance relies on large-scale annotated data that can adequately represent the complex conditions of forests; second, the inherent low overlap, point density variation, vegetation occlusion, and instability of geometric features in forest scenes severely limit the generalization ability of current deep learning models, causing the final registration results to often fail to meet practical application needs [12,14,24].

Currently, most of existing forest point cloud registration studies are based on single-platform or single-view point cloud data [34]. Datasets such as KITTI [35], 3Dmatch [36], ETH [37], and WHU-TLS [38] focus on structured indoor or urban outdoor scenes and are limited to single-platform data, unable to support multi-platform registration and cross-view registration tasks. Forest-specific point cloud datasets, such as FOR Instance [39], Forest Semantic [40], International TLS Benchmarking [41], NEWFOR Benchmark Dataset [42] and Broadleaf forests [43], primarily focus on tasks tree segmentation and forest inventory. Chen et al. [34] have provided the first multi-platform forest point cloud registration benchmark dataset, covering 17 sample plots and 1.56 billion points, offering a basic testing platform for multi-platform point cloud registration. The dataset is restricted to foundational forest scenarios represented by ULS and TLS data, and does not encompass specialized datasets for specific economic forests, such as rubber plantations. Additionally, it omits the use of emerging lightweight platforms like ULS and HLS. Furthermore, the dataset neglects seasonal variations and is devoid of airborne point cloud data representing rubber trees at various growth stages.

In this paper, a registration method for complex HLS-ULS rubber tree canopies is presented, which has proven to be effective and robust in experiments across diverse cross-view plots:

(1) A semantics-guided, automatic registration method is proposed for rubber tree point clouds. This pose-free approach is particularly effective for cross-view registration in weakly structured scenarios, and has been successfully evaluated with high accuracy.
(2) The method introduces a Woody Salient Keypoint (WSK) extraction algorithm that rapidly identifies keypoints with high repeatability, viewpoint invariance, and favorable spatial distribution through semantic-guided Regions of Interest (ROI) segmentation and salient point detection. It can still extract representative point primitives in rubber plantations with weak structural features and high local repetitiveness.
(3) A benchmark ULS-HLS dataset for rubber trees has been released. It covers 25 sample plots across 5 testing areas, comprising over 400 million points. The dataset includes data from both leaf-on and leaf-off seasons, facilitating research on structural parameter extraction and phenotyping of rubber trees.

The remainder of this paper is organized as follows. Section 2 introduces the materials and methods, including the LiDAR platforms, study area, dataset, and the proposed co-registration method. Section 3 presents the experimental results, covering parameter sensitivity, registration performance, and comparative analysis. Section 4 discusses the findings, focusing on seasonal impacts and method generalization. Finally, Section 5 concludes the paper.

## 2. Materials and Methods

### 2.1. UAV and Handheld LiDAR Platforms

ULS data were acquired using a DJI Matrice 300 UAV (Shenzhen DJI Sciences and Technologies Ltd., Shenzhen, China) equipped with a CBILite LiDAR scanner (Chengdu Orenda Technology Co., Ltd., Chengdu, China), featuring a 360° horizontal field of view and a 31° vertical field of view. The system has a maximum measurement range of 120 m, with a distance accuracy of 5 cm at 70 m and an absolute positioning accuracy of approximately 10 cm. The flight altitude was maintained at 80 m, with a flight speed of 4 m/s. HLS data were collected using the LiGrip H120 handheld system (GreenValley International, Beijing, China), which provides a 360° horizontal field of view and a 280° vertical field of view, with a measurement range of 120 m and a distance accuracy better than 5 cm. This platform was primarily used to supplement and validate ULS data in registration experiments. Both the ULS and HLS systems use the industry-standard 905 nm laser wavelength, which is widely applied in lightweight, cost-effective commercial LiDAR systems, balancing eye safety and hardware affordability. The parameters of the equipment are presented in Table 1.

### 2.2. Study Sites and Dataset

**Figure 1:**
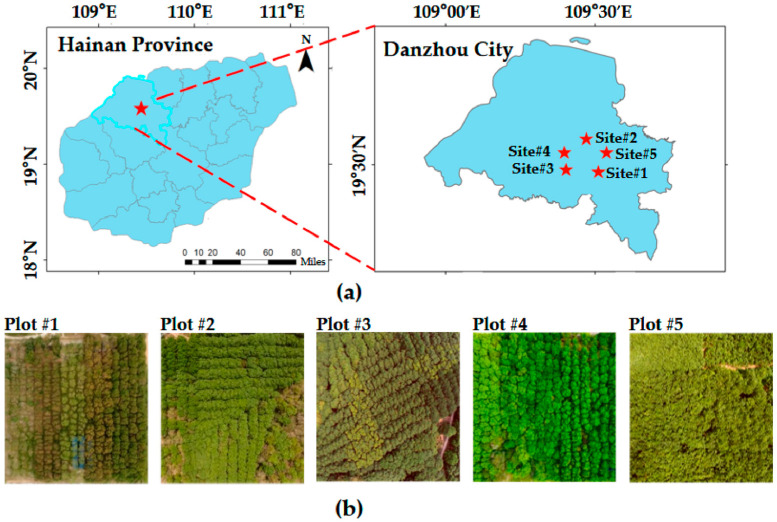
Overview of the rubber plantation research area; (**a**) Geographical location of the rubber plantation in Danzhou city; (**b**) Field photos of the study area.

The study was conducted in five rubber plantations (as shown in Figure 1) located in Danzhou City, Hainan Province, China (19°19′ N, 109°20′ E). This region was selected as the data collection area for ULS and HLS. The dataset used for the registration of HLS and ULS was sourced from all five rubber plantations (as shown in Figure 1), including Jinfu Rubber Plantation (Site #1), Hongcheng Rubber Plantation (Site #2), Sidui Rubber Plantation (Site #3), Jiudui Rubber Plantation (Site #4), and Miaopu Rubber Plantation (Site #5). Five systematic 30 m × 30 m sample plots were established within each rubber plantation survey area to evaluate the method’s robustness under different conditions and to verify the statistical representativeness of the sampling.

Significant differences exist in tree structures, seasonal canopy complexity, and the growth stages of rubber trees across the survey areas. Sites #1–#4 representing mature rubber plantations, and Site #5 representing a young rubber plantation, with notable variations in tree planting density. The data collection covers different seasonal periods, with Sites #1, #2, #3, and #5 representing incomplete leaf-off period data, and Site #4 representing leaf-on period data. The point cloud characteristics were governed by the extent of leaf coverage, which varied substantially across different growth periods. Detailed forest stand parameters for all Sites are provided in Table 2. Furthermore, the point cloud data of the dataset has been rigorously manually registered to ensure precise alignment of multi-view point clouds, providing a reliable benchmark for the registration validation.

The difficulty levels of the survey Sites were evaluated, with the results presented in Table 2. The difficulty classification criteria primarily considered station spacing, point cloud overlap, canopy closure, and leaf growth conditions. Specifically, Sites #1, #2, #3, and #5 were classified as low difficulty due to sparse foliage, which enhanced the penetration capability of ULS point clouds and preserved more structural details. In contrast, Site #4 was classified as high difficulty due to dense foliage and severe occlusion, resulting in point cloud gaps and increased heterogeneity between platforms.

### 2.3. Tree Keypoint Matching for Cross-View Registration

**Figure 2:**
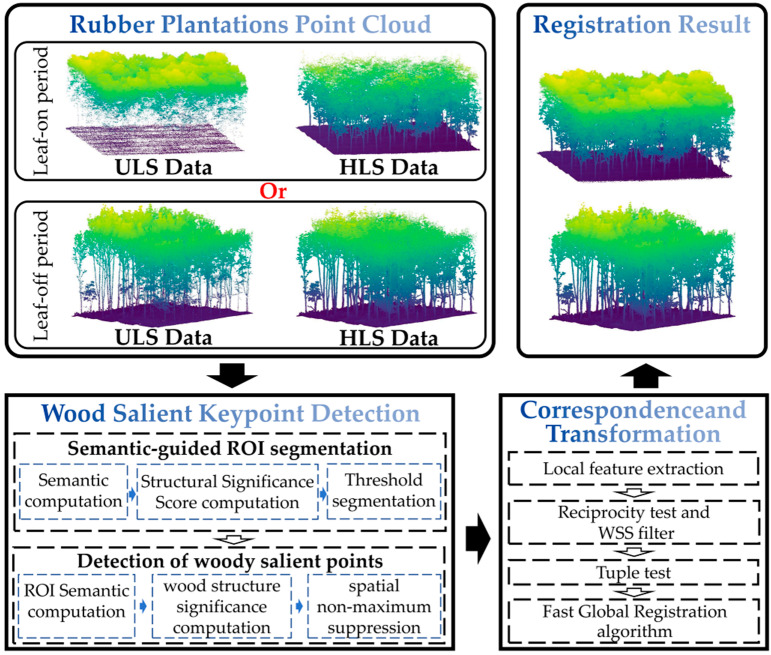
Overview of the proposed method for registering ULS-HLS rubber tree forest point clouds.

This paper proposes a novel lightweight preprocessing method for point cloud registration, specifically designed for HLS-ULS cross-view registration tasks. The core innovation lies in the development of a keypoint extraction method that integrates semantic-guided ROI segmentation with the detection of woody salient points. This approach leverages semantic information to efficiently extract structurally stable ROIs and directly detects keypoints with high distinctiveness and viewpoint consistency based on these ROIs. Building upon these keypoints, local feature description, the reciprocity test, the tuple test, and Fast Global Registration (FGR) [44] are integrated to achieve high precision. The overall workflow is illustrated in Figure 2. The proposed algorithms were implemented in C++ using the Point Cloud Library (PCL). The specific hardware configuration used for all runtime evaluations is detailed in Section 3.

#### 2.3.1. Woody Salient Keypoint Detection

To enhance the robustness of ULS-HLS data registration, a unified strategy combining semantics-guided region segmentation and WSK detection is proposed. The objective is to suppress unstable foliage regions, concentrate computational resources on structurally stable woody components, and extract keypoints that are both distinctive and repeatable across viewpoints. In terms of semantic feature construction, we use the covariance matrix (Equation (1)) to represent the local geometric attributes of each point. Through extensive experiments, we found that linearity (*L*), the first principal component (*PCA1*), and Surface variation (*Sv*) (Equation (2)) yield the best results when characterizing woody regions. Therefore, we constructed the Structural Significance Score (*SSS*) (Equation (3)) based on these features. Specifically, the covariance matrix is defined as follows:

(1) $$C_{3\times 3}=\frac{1}{N}\sum_{i=1}^{N}(p_{i}-\mu ){\left(p_{i}-\mu \right)}^{T}$$

where *C*_3×3_ represents the covariance matrix, *p_i_* denotes the *i*-th point in the neighborhood *R* of the reference point, *μ* is the geometric centroid of the neighborhood, and *N* is the total number of points in the neighborhood. Here, the neighborhood radius *R* is set to 0.5 m. Subsequently, the covariance matrix undergoes Singular Value Decomposition (SVD) to obtain three eigenvalues *λ*_1_, *λ*_2_, *λ*_3_, where *λ*_1_ *> λ*_2_ *> λ*_3_. The semantic features are computed by Equation (2).

(2) $$\begin{array}{ccc} L=\frac{\lambda_{1}-\lambda_{2}}{\lambda_{1}}, & PCA1=\frac{\lambda_{1}}{\lambda_{1}+\lambda_{2}+\lambda_{3}}, & Sv=\frac{\lambda_{3}}{\lambda_{1}+\lambda_{2}+\lambda_{3}} \end{array}$$

The SSS is calculated using the following formula:

(3) SSS=L+PCA1−Sv

**Figure 3:**
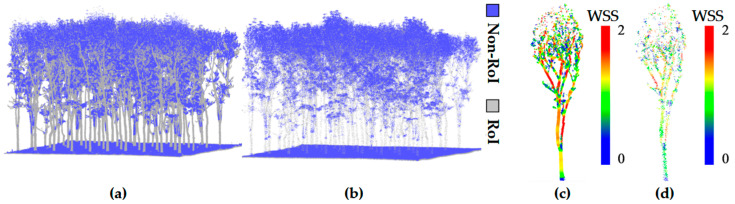
ROI Segmentation and WSS Significance Distribution of HLS and ULS Point Clouds: (**a**,**b**) show the ROI segmentation results for HLS and ULS point clouds, respectively; (**c**,**d**) depict the WSS significance distribution within the ROI for HLS and ULS point clouds, respectively.

After constructing the semantic features, the point cloud is quickly segmented into ROI and Non-Region of Interest (non-ROI) based on a set threshold *τ*. Figure 3 shows the segmentation results of the point cloud in these two regions:

- ROI: Regions with strong structural stability and geometric significance (such as woody components), which exhibit consistency from different viewpoints and are suitable as the foundation for keypoint extraction and registration.
- Non-ROI: Regions with unstable geometric structures (such as leaf noise), which are easily affected by external disturbances, making it difficult to ensure the reliability of keypoints, and thus unsuitable as the basis for keypoint extraction.

After the rapid segmentation stage, WSK detection is performed within the ROIs to extract highly discriminative keypoints from structurally stable regions. The proposed method relies on structural complexity metrics to accurately localize points with locally unique geometry while effectively suppressing redundant information. The core process consists of two steps:

First, candidate points are sorted and filtered based on the WSS score. The wood structure significance (WSS) value for all points in the ROI is calculated (Equation (4)) to quantify the structural complexity of the local neighborhood. A lower value indicates a more unique structure (e.g., branch bifurcation points). The points are then sorted in ascending order based on their WSS values, and the top *N_r_* candidate points are selected. The WSS structural significance expression is defined as follows:

(4) $$WSS=\frac{L+(1-Sv)}{1+\ln(1+P+S)}$$

where *L* represents linearity, *Sv* denotes curvature, *P* represents planarity, and *S* denotes scatter, where the calculation formula for linearity has been given in Equation (2). The definitions of the other semantic features are as follows:

(5) $$\begin{array}{cc} P=\frac{\lambda_{2}-\lambda_{3}}{\lambda_{1}}, & S=\frac{\lambda_{1}}{\lambda_{3}} \end{array}$$

Finally, spatial non-maximum suppression (NMS) is applied to the selected candidate points to address the issue of spatial clustering, ensuring that the keypoints are evenly distributed in space. Specifically, within the local neighborhood of each point, only the point with the lowest WSS value (i.e., the most structurally unique) is retained, suppressing the points with relatively lower complexity around it. The complete keypoint extraction process is shown in Algorithm 1.
**Algorithm 1** Woody Salient Keypoint Detection **Input:** Forest point cloud: *P_F_***Output:** The WSK: *K_W_*1.**Procedure:** ROI Segmentation2.Initialization: ROI set *P_I_* ← *ϕ*3.    **For** each point *p_i_* ∈ *P_F_* do4.        Compute *SSS* via Equation (3)5.        **If** *SSS* > *δ* then6.              *P_I_* ← *P_I_* ∪ {*p_i_*}7.        **End if**8.    **End for**9.**End Procedure**10.**Procedure:** Keypoints Detection11.**Initialization:** feature set *F* ← *ϕ*, point set *K_I_* ← *ϕ*12.    **For** each point *p_i_* ∈ *P_I_* do13.          Compute *WSS* via Equation (4)14.          *F* ← *F* ∪ {(*p_i_*, *WSS*)}15.    **End for**16.    Sort *F* in ascending order by *WSS*.17.    *K_I_* ← The top *Nr* most significant points.18.**Initialize:** keypoint set *K_W_* ← *ϕ*, suppression map set *M* ← {false}19.    **For** each point *p_i_* ∈*K_I_* do20.        **If** *m_i_* = false then21.            **If** *WSS_pi_* < *WSS_pi-neighbors_* then22.                *K_W_* ←*K_W_* ∪ {*p_i_*}, *m_pi-neighbors_* ← true23.            **Else** *m_pi_* ← true24.            **End if**25.        **End if**26.    **End for**27.**End Procedure**28.**Return** WSK set *K_W_*

#### 2.3.2. Correspondence and Transformation Estimation

Following WSK detection, a feature descriptor with high distinctiveness, noise robustness, and density adaptability is therefore needed to address the challenges of structural similarity and occlusion in forest point clouds. To this end, a 352-dimensional Signature of Histograms of OrienTations (SHOT) descriptor [45] is employed to encode the local geometric structure around each keypoint. Specifically, for the source keypoints *p* ∈ P and the target keypoints *q* ∈ Q, feature representations F(*p*) and F(*q*) are extracted, respectively. For each point *p* ∈ P, the nearest neighbor of F(*p*) is searched in F(Q); similarly, for each point *q* ∈ Q, the nearest neighbor of F(*q*) is searched in F(P). These correspondences form the initial matching sets *C_P_* and *C_Q_*. Due to partial overlap, non-uniform density, and repetitive structures (e.g., similar woody components among different trees), these initial sets often include numerous incorrect matches. To refine the correspondences, two sequential strategies are applied:

First, reciprocity testing is performed in conjunction with a WSS saliency constraint. For any *p*_*i*_ ∈ *P*, a correspondence with *q*_*j*_ ∈ *Q* is retained if:

(6) $$\left\{\begin{array}{c} p=\underset{p_{i}\in P}{\arg\min(Dis(F(q),F(p_{i})))}\\ q=\underset{q_{j}\in Q}{\arg\min(Dis(F(p),F(q_{j})))}\\ \left|w(p)-w(q)\right|<\delta \end{array}\right.$$

the correspondence pair (*p*, *q*) is retained. All correspondences satisfying Equation (6) constitute the set K*_II_*, δ is set to 0.3.

Second, a tuple consistency test is performed: three correspondence pairs are randomly sampled from *K_II_*, denoted as (*p*_1_,*q*_1_), (*p*_2_,q_2_), and (*p*_3_,*q*_3_). The compatibility of these tuples is verified by checking whether the triplets (*p*_1_, *p*_2_, *p*_3_) and (*q*_1_, *q*_2_, *q*_3_) satisfy the following geometric consistency condition:

(7) $$\begin{array}{ccc} \forall i\neq j, & \alpha \leq \frac{\left\|p_{i}-p_{j}\right\|}{\left\|q_{i}-q_{j}\right\|}\leq \frac{1}{\alpha }, & 0<\alpha <1 \end{array}$$

where *α* is a tolerance parameter. The correspondences passing this test are retained to form the refined set *K_III_*, which is subsequently used for global transformation estimation.

Finally, the rigid transformation aligning the source and target point clouds is estimated from *K_III_* using the FGR algorithm. Finally, the rigid transformation to align the source and target point clouds is estimated from *K_III_* using the FGR algorithm, which efficiently computes the optimal alignment while mitigating the impact of outliers.

#### 2.3.3. Evaluation Criteria

To evaluate the accuracy of the proposed registration method, this paper introduces a reference point cloud *P*^∗^ and performs a quantitative comparison with the registration results. *P*^∗^ is obtained by manually aligning the point cloud multiple times and precisely selecting a large number of corresponding points, ensuring optimal alignment of the point cloud from multiple viewpoints. It can be indirectly considered as “ground truth” data.

In the evaluation phase, three core evaluation metrics are designed to measure the global alignment between the two point clouds: the three-dimensional principal axis angle (*θ*), centroid Euclidean distance (*D*_*centroid*_), and Root Mean Square Error (RMSE). For the three-dimensional principal axis angle (*θ*), it is calculated based on Principal Component Analysis (PCA) to measure the angular deviation between the principal axis vectors of *P*^∗^ and the registered transformed point cloud *P*′. This metric quantifies the overall three-dimensional rotational error of the registration:

(8) $$\theta =\arccos\left(\frac{V^{*}\cdot V^{\prime }}{\left\|V^{*}\right\|\cdot \left\|V^{\prime }\right\|}\right)\cdot \frac{180}{\pi }$$

where *V*^∗^ and *V*′ represent the principal axis direction vectors of the reference point cloud and the transformed point cloud, respectively. Additionally, spatial consistency is measured by the Euclidean distance between the centroids of *P*^∗^ and *P*′, which reflects the overall translational error. The calculation formula is as follows:

(9) $$D_{Centroid}=\left\|C_{S}-C_{T}\right\|$$

Finally, to assess the precision of the registration results at the local point level, the RMSE is introduced as a statistical metric for the point-wise positional deviation. It is computed on the set of valid corresponding points, and the formula is as follows:

(10) $$RMSE=\sqrt{\frac{1}{N}\sum_{i=1}^{N}{\left\|p_{i}^{*}-p_{i}^{\prime }\right\|}^{2}}$$

where *N* is the number of points in the point cloud, and $p_{i}^{*}$ and $p_{i}^{\prime }$ represent the points in the reference point cloud and the transformed point cloud, respectively.

## 3. Results

All algorithms were implemented in C++ using the PCL. The performance evaluations, including all execution times reported subsequently, were conducted on a Windows 11 workstation (Lenovo Group Ltd., Beijing, China) equipped with an AMD Ryzen 7 3700X CPU and 16 GB of RAM to ensure consistency.

### 3.1. Parameter Setting and Sensitivity Analysis

**Figure 4:**
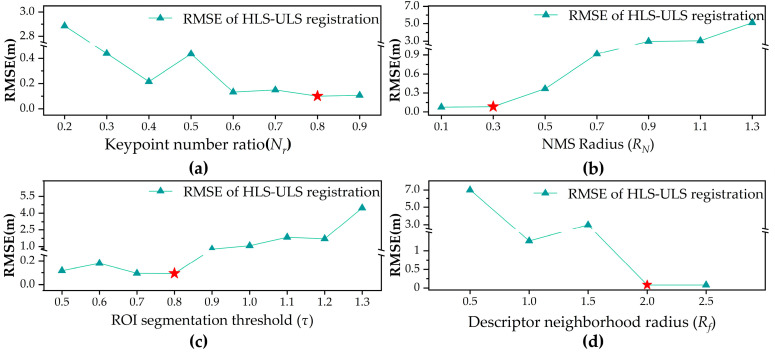
Parameter Sensitivity Analysis. (**a**) Distribution of registration accuracy under different segmentation thresholds. (**b**) Distribution of registration accuracy under different keypoint retention ratios. (**c**) Distribution of registration accuracy under different conditions. (**d**) Relationship between descriptor neighborhood radius and RMSE. The five-pointed stars mark the optimal parameter values identified in each analysis.

In this study, the parameters were determined through analysis of the experimental dataset and optimized via multiple iterations, as shown in Figure 4. Unless otherwise specified, a unified parameter configuration was used for ULS and HLS point cloud registration. To reduce computational load, memory usage, and mitigate point cloud density differences caused by varying acquisition angles between ULS and HLS, a 0.15 m uniform grid sampling method was used during preprocessing.

The key parameters of the proposed registration method include the keypoint number ratio (*N_r_*), the NMS radius (*R_N_*), the ROI segmentation threshold (*τ*), and the feature extraction radius for descriptors (*R_f_*). To systematically assess the independent impact of each parameter on registration performance, a one-factor sensitivity analysis was employed: other parameters were fixed, and only the target parameter was adjusted to quantify the accuracy changes induced by this adjustment.

The *N_r_* represents the proportion of points with the highest structural significance based on the WSS. If *N_r_* is too small, key structural information may be lost, reducing registration accuracy and robustness. Conversely, if *N_r_* is too large, redundant information may be introduced, increasing computation time and the risk of mismatches between highly similar woody structures, which is particularly prominent in monoculture rubber plantations. As shown in Figure 4a, in HLS-ULS registration, RMSE decreases as *N_r_* increases, stabilizing at *N_r_* = 0.6, with the lowest error at *N_r_* = 0.8. Therefore, *N_r_* is set to 0.8 for HLS-ULS.

The *R_N_* is the support radius used in the NMS operation during keypoint detection, controlling the spatial distribution density of keypoints. As shown in Figure 4b, as *R_N_* increases, keypoint numbers decrease, leading to loss of structural information and increased registration error. For HLS-ULS, RMSE decreases as *R_N_* increases, reaching a minimum at 0.3 m, after which it rises. *R_N_* is set to 0.3 m. This optimal radius is designed to promote a well-distributed spatial arrangement of keypoints on stable woody structures, preventing excessive clustering on the woody framework, which is crucial for capturing local structural features of keypoints in subsequent steps.

The *τ* parameter is used to segment the point cloud based on the SSS, distinguishing ROI from Non-ROI. Adjusting the *τ* value enables the regulation of the size and quantity of the segmented ROI regions. If *τ* is set too low, woody regions that should be retained in the rubber tree point cloud may be excluded, leading to the loss of critical structural information, disrupting spatial correspondences between keypoints, or indirectly resulting in insufficient keypoints, thereby reducing the robustness of the registration process. In contrast, if set excessively high, it may incorporate an overabundance of irrelevant points, thereby reducing keypoint stability, increasing mismatches, augmenting the computational load, and potentially compromising registration accuracy. A reasonable setting excludes irrelevant points and retains stable, structurally rich ROIs, improving accuracy and reducing mismatches. As shown in Figure 4c, for HLS-ULS, *τ* values between 0.7 and 0.8 give stable performance. In light of these results, *τ* is set to 0.8.

The *R_f_* is the neighborhood radius for constructing the SHOT descriptor, affecting both its discriminative ability and computational efficiency, and is intended to capture local structural features that distinguish different tree trunks or main branches. A small *R_f_* limits information, while a large *R_f_* reduces efficiency and robustness. Based on dataset characteristics, *R_f_* is set to 2.0 m for local feature extraction.

### 3.2. Registration Performance

**Figure 5:**
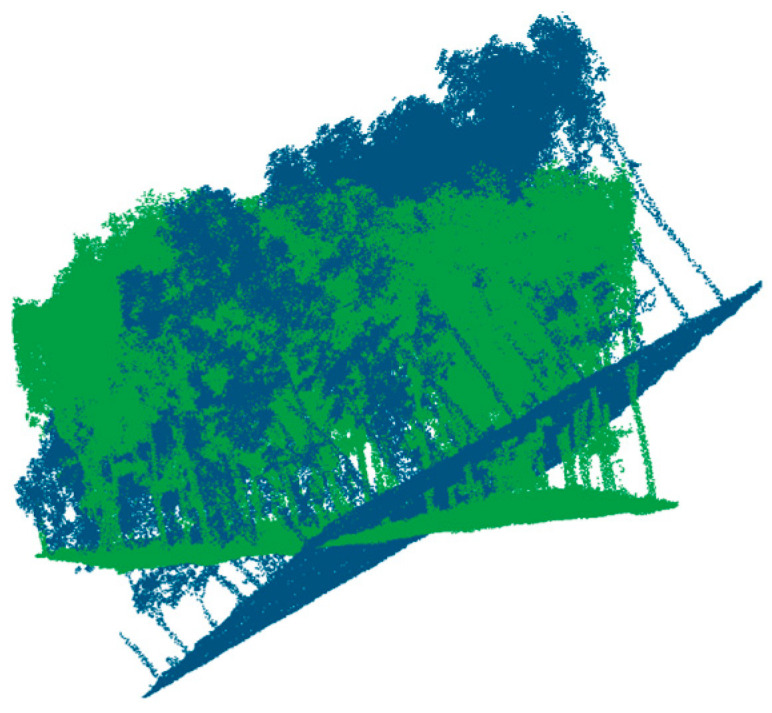
Initial spatial state of ULS (blue) and HLS (green) point clouds before registration.

Figure 5 shows the erroneous spatial distribution of the point clouds after applying the same predefined initial transformation across all survey sites. Owing to the internal consistency of samples within each site, one plot per survey area was selected to display the pre-registration point clouds. Overall, the proposed method is shown to enable robust registration between HLS and ULS point clouds across different sites and time periods.

**Figure 6:**
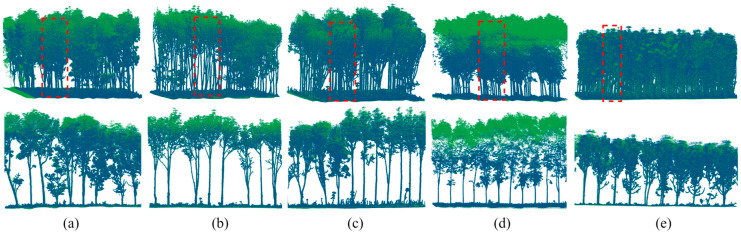
Registration results and details of HLS (blue) and ULS (green) point clouds in representative plots are presented. The red boxes indicate the cropped areas of local cross-sections. (**a**–**e**) show the overall (**upper**) and local (**lower**) registration results.

Figure 6 illustrates the registration results of HLS–ULS point clouds and Table 3 presents the average registration results for the five sample Sites used for HLS–ULS point cloud registration within each survey area. The overall RMSE across all five measurement Sites is 0.09 m, with Site #1 performing the best, achieving an RMSE of 0.06 m. This may be due to the sparse foliage of the trees, resulting in lower heterogeneity between point clouds. The registration time for all Sites ranges from 2.93 to 4.35 min, with an average of 3.49 min. Furthermore, the experiment shows that when higher registration efficiency is required, the number of candidate matching points can be reduced by appropriately decreasing parameters *N_r_* and *τ*, thereby shortening the registration time. However, this may slightly reduce the robustness and final registration accuracy of the method.

### 3.3. Efficacy Evaluation of the Keypoint Module

**Figure 7:**
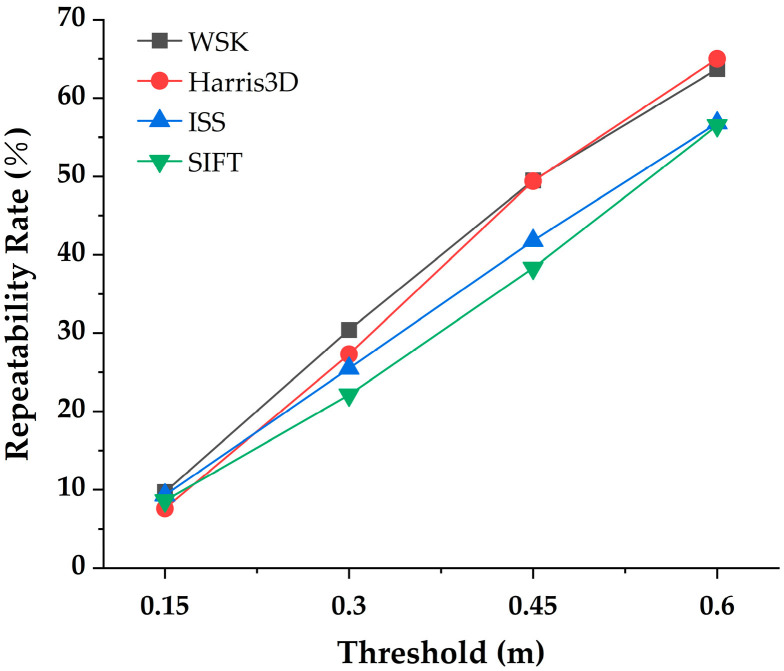
Keypoint repeatability across viewpoints at different thresholds.

To assess the effectiveness of the proposed WSK detector in forest point cloud registration, it was compared with three widely used keypoint detectors (ISS, Harris3D, and SIFT). In all experiments, the NMS radius was uniformly set to 0.3 m, and thread acceleration was not applied. The repeatability of the four detectors was systematically compared, and their performance was evaluated on representative sample plots in seven different regions. Figure 7 illustrates the performance of each keypoint detector under different distance thresholds. At lower thresholds (0.15 m and 0.3 m), WSK demonstrated significant advantages. The higher repeatability at smaller distances plays an important role in improving the accuracy and stability of subsequent point cloud registration. Specifically, WSK’s repeatability was noticeably higher than that of the other detectors within the smaller threshold range (0.15 m to 0.3 m), and as the threshold increased (0.45 m to 0.6 m), the repeatability of all detectors improved, but WSK still performed well.

To further evaluate the performance differences in the WSK algorithm in forest point cloud registration, particularly in terms of keypoint extraction efficiency and subsequent registration accuracy, ablation experiments were conducted. In these experiments, the number of keypoints for all methods was fixed at the same order of magnitude for comparison. The nearest neighbor distance of each keypoint in the source point cloud was calculated and averaged to assess the distribution of keypoints in the point cloud. The keypoint extraction time was also recorded and applied to subsequent registration tasks. Table 4 displays the number of keypoints, Average Nearest Neighbor Distance (ANND), keypoint extraction time, and registration RMSE for the four methods. The number of keypoints extracted by WSK was moderate. The average nearest neighbor distances for all methods were similar, with ISS showing the lowest distance (0.34 m), while WSK and SIFT had values of 0.37 m and 0.39 m, demonstrating good point cloud distribution characteristics. In terms of computational efficiency, WSK performed well, where its keypoint extraction time was 7 s, significantly shorter than SIFT’s 34.5 s, while Harris3D and ISS took 3.6 s and 3 s, respectively. Although the extraction time for WSK was slightly longer than that of ISS and Harris3D, its extraction efficiency remained competitive.

Regarding registration accuracy, WSK demonstrated outstanding performance. WSK’s registration RMSE was 0.06 m, significantly outperforming Harris3D and SIFT. This indicates that WSK not only provides high-quality keypoint extraction but also maintains high accuracy in subsequent point cloud registration. Additionally, despite ISS having a shorter keypoint extraction time, its registration RMSE was 0.07 m, slightly better than SIFT, but still lower than WSK. WSK demonstrated its unique advantage in providing high repeatability and low RMSE, proving its potential application in cross-view point cloud registration for forest scenes.

### 3.4. Comparison Experiments

This study compares two classic point cloud registration methods: one based on downsampled keypoints combined with Fast Point Feature Histogram (FPFH) and Random Sample Consensus (SAC-IA) registration [46], which is widely applied in the field of point cloud registration due to its efficiency and robustness, making it a classic solution. The second is a state-of-the-art deep learning-based method, HregNet [28]. It performs registration by extracting and matching multi-level keypoints and descriptors, utilizing bilateral and neighborhood consensus mechanisms to generate accurate point correspondences. In comparison to traditional methods, HRegNet takes advantage of deep learning networks, improving matching accuracy in complex scenes, particularly in the stability of feature extraction and matching.

**Figure 8:**
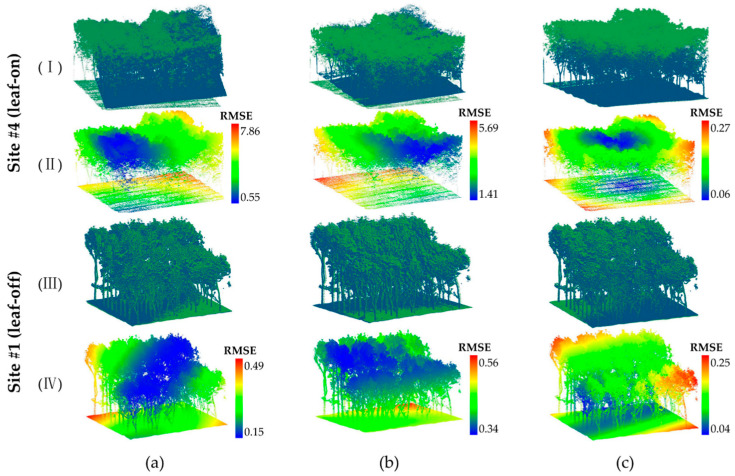
Performance comparison of point cloud registration methods under different seasonal conditions. (**a**) SAC-IA method; (**b**) HRegNet method; (**c**) Our method. The HLS and ULS point clouds are colored blue and green, respectively. The subfigures labeled with Roman numerals represent: (**I**) leaf-on season registration results; (**II**) leaf-on season registration error visualization; (**III**) leaf-off season registration results; (**IV**) leaf-off season registration error visualization.

Based on the quantitative comparison results presented in Table 5, our method demonstrates superior performance across all test scenarios, with a maximum registration RMSE of 0.14 m and a minimum RMSE of 0.06 m, showing stable performance. The SAC-IA method exhibits highly unstable performance across different Sites, with a maximum registration RMSE of 12 m. This may be due to the lack of structural awareness in the downsampled keypoints, which results in the introduction of many similar features during subsequent feature extraction and matching, leading to registration instability. In forest scenes, where trees exhibit many similar local features, ambiguity in feature matching may arise. Methods such as HRegNet rely on the extraction and matching of feature points; however, when the geometric features of the scene are similar or repetitive, the matching algorithms may be interfered with, resulting in reduced matching accuracy. Focusing on the most representative seasonal cases (leaf-on for Site #4 and leaf-off for Site #1), Figure 8 provides visual results consistent with the comprehensive quantitative evaluation in Table 5. Our method achieves continuous, tight alignment in both seasons (subfigures c-I and c-III) with minimal error (blue areas in c-II and c-IV). In contrast, SAC-IA (a) and HRegNet (b) show noticeable misalignments, especially under the complex leaf-on conditions (a-I, b-I), which correspond to their higher RMSE values in Table 5.

## 4. Discussion

### 4.1. Impact of Seasonal Structural Differences

Seasonal changes in rubber plantations, specifically the distinct phenological shift from leaf-on to incomplete leaf-off conditions, fundamentally alter LiDAR point clouds’ density distribution and geometric features from both aerial and ground perspectives [47,48]. The performance of cross-platform registration is consequently affected, as these seasonal alterations influence keypoint detectability, the stability of feature matching, and, ultimately, the overall registration accuracy.

**Figure 9:**
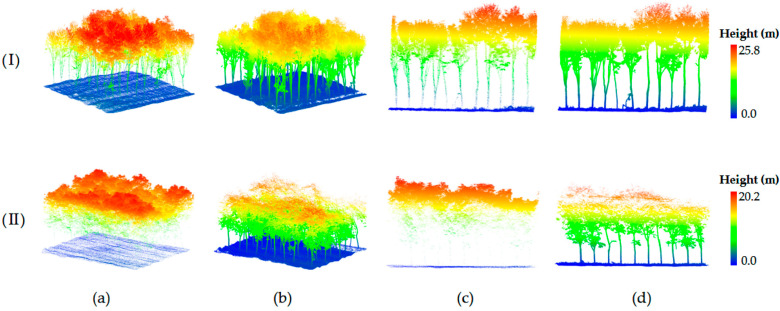
Point cloud data across seasons. (**Ι**) and (**ΙΙ**) correspond to incomplete leaf-off and leaf-on point clouds, respectively; (**a**,**b**) show HLS and ULS point clouds; (**c**,**d**) present local slices of the HLS and ULS point clouds.

Figure 9 shows the spatial distribution of point clouds from the rubber plantation during the leaf-on and leaf-off seasons. During the leaf-on season, the tree leaves and branches are lush, with ULS point clouds predominantly concentrated in the canopy, offering a detailed depiction of the tree crown. However, its penetration capability into the lower tree trunks and the ground region is significantly reduced [49]. In contrast, while HLS perspective clearly captures tree trunks and ground information, it fails to express the canopy adequately. In the incomplete leaf-off season, the shedding of leaves weakens the occlusion effect, resulting in a more continuous vertical distribution of LiDAR point clouds from both aerial and ground platforms [50]. This enhanced continuity and consistency in the vertical structure provide a richer common view for registration. Thus, seasonal changes, by altering vegetation occlusion and point cloud spatial distribution, significantly enhance or reduce the structural heterogeneity across platforms, directly affecting the stability and reliability of the initial matching phase in registration algorithms.

**Figure 10:**
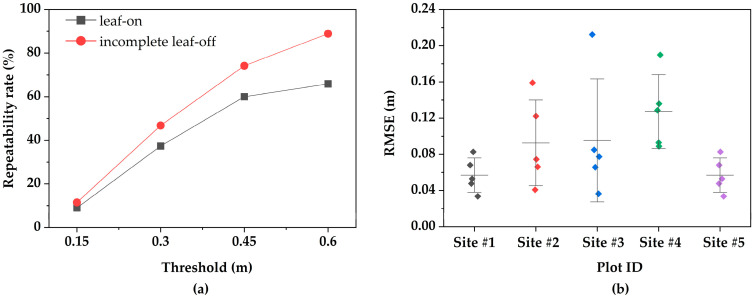
Keypoint Repeatability and Registration Accuracy Across Different Seasons. (**a**) Keypoint repeatability rate during the leaf-on and partial leaf-off seasons. (**b**) Registration accuracy of HLS-ULS point clouds across different sites, with Site #4 representing leaf-on point cloud data and the others representing incomplete leaf-off point cloud data.

Seasonal variation, by modifying the morphological structure of forests, directly impacts the geometric features and spatial continuity of the point clouds, presenting challenges for the stability of keypoint detection and matching. To quantitatively assess the robustness of the WSK detector under different seasonal conditions, we analyzed the repeatability of keypoints during the leaf-on and incomplete leaf-off seasons, as shown in Figure 10a. The results indicate a significant difference in keypoint matching rates between the two seasons under different radius thresholds, with WSK demonstrating a notably higher matching success rate during the leaf-off season. At smaller radius thresholds (0.15 m and 0.3 m), the matching rates during the leaf-off season (11.5% and 46.8%) were substantially better than those during the leaf-on season (9% and 37.4%). This can largely be attributed to the clearer and more stable geometric form of the exposed woody structures after leaf shedding, enabling the WSK to extract keypoints with higher positional accuracy and repeatability, such as branch bifurcation points [51]. During the leaf-on season, despite the dense foliage introducing significant geometric noise, reducing the spatial continuity of the point clouds, and exacerbating the heterogeneity of point cloud data from both ground and aerial platforms [52], WSK’s unique woody structure significance detection mechanism still enables stable extraction of keypoints centered on tree trunks and primary branches, even from partially occluded and noisy point clouds.

Furthermore, we evaluated the cross-platform registration accuracy under different seasonal conditions. Figure 10b presents registration results from several sample Sites, including RMSE, where each data point represents the average value obtained from multiple independent registration experiments for the corresponding site. The results show that the registration accuracy during the leaf-off season generally outperforms that of the leaf-on season. During the leaf-off season, after the leaves have fallen and the woody structures are exposed, the point cloud data becomes cleaner and more stable. The keypoints extracted by WSK exhibit higher repeatability and matching stability, significantly improving registration accuracy. This is mainly due to the high-quality matching sets formed during the leaf-off season, providing abundant and accurate correspondences for FGR optimization, thereby making the rigid transformation estimation more robust. The larger errors in some sample sites may stem from poor point cloud quality, resulting in fewer stable features for the algorithm to rely on, thus increasing registration errors. In contrast, the error in leaf-on season sample Sites (e.g., Site #4) was noticeably larger. This phenomenon can be attributed to the lower keypoint matching rate under leaf-on conditions. The occlusion caused by leaves and branches leads to a decreased proportion of inlier points in the initial matching. Although subsequent reciprocity testing and tuple consistency filtering eliminate most erroneous matches, the insufficient number of reliable matches increases the uncertainty of the transformation estimation and reduces registration accuracy.

In summary, the systematic analysis of point cloud distribution, keypoint matching, and registration results reveals the full impact chain of seasonal changes on cross-platform registration in forest scenes. The clear and geometrically consistent point cloud structure during the leaf-off season leads to higher stability and precision in keypoint detection and matching by the WSK detector, resulting in excellent registration outcomes. However, the complex structure and severe occlusion during the leaf-on season remain significant challenges for cross-view forest point cloud registration.

### 4.2. Performance on Other Tree Species

To evaluate the applicability and generalization ability of our method in broader forest environments, we applied it to a multi-region, multi-species point cloud registration public benchmark dataset [34]. This dataset covers 7 regions and 17 plots, containing over 1.5 billion points, with significant diversity in point cloud area, density, terrain, stand structure, and tree species (including mixed forests, willow, poplar, white birch, larch, fir, and others). Based on this, this section focuses on evaluating the method’s generalization ability and robustness across these quantifiable structural key factors. As shown in Table 6, the method demonstrates stable performance in most sites, though larger registration errors were observed in sites S01, S02, and S09. For cases with larger errors, fine registration algorithms, such as Iterative Closest Point (ICP) [53], could be introduced to refine the pose estimation.

Error analysis reveals that point cloud density is a key factor influencing registration accuracy [18]. Sites S02 and S09, with lower point cloud densities, resulted in insufficient representation of tree geometry and details, thereby increasing matching difficulty. Although S01 has a relatively higher point cloud density, it was still affected by high tree density and canopy occlusion. This limited the ULS point cloud’s ability to capture tree trunks and lower structures, which constrained the accuracy of keypoint matching and feature description.

In contrast, despite sparse ULS point clouds in S04, the woody structures were still effectively represented, supporting stable registration. S10, characterized by a sparse forest stand and low canopy closure, exhibited high structural integrity, leading to good registration performance. In S11 to S14, although the ULS point cloud densities were not high, the dominance of coniferous trees weakened the canopy occlusion effect, allowing for better representation of tree geometry. Thus, even with similar point cloud densities, coniferous forests’ point cloud data was able to more effectively capture structural information, particularly the geometric forms of tree trunks and branches, improving both registration and feature extraction performance [54,55]. In comparison, broadleaf forests with dense crowns and overlapping leaves are prone to significant canopy occlusion, which hinders the effective capture of structural information from lower wood and tree trunks, thus reducing registration accuracy [56,57].

In summary, the method proposed in this study exhibits good robustness across diverse environments. The analysis results indicate that point cloud density, tree distribution density, and tree species type significantly affect registration accuracy within this dataset. Higher point cloud density aids in accurately capturing structural details, enabling the construction of strong correspondences in WSK and effective feature extraction, reducing registration errors. In areas with higher tree density, the smaller spacing between trees worsens canopy occlusion, leading to sparse geometric information in the point cloud and making it difficult to fully express tree structures, which increases the difficulty of point cloud matching. Coniferous forests, due to their typically conical shape and weaker canopy occlusion, achieve high registration accuracy even at lower point cloud densities, whereas broadleaf forests, with their complex canopy structure and occlusion effects, present greater challenges for registration.

### 4.3. Limitations and Future Work

The registration method proposed in this study demonstrates a significant advantage in terms of accuracy, its coverage across the growth stages of rubber trees remains discontinuous. Subsequent research will focus on its cross-stage application across different growth periods of rubber trees, aiming to develop a dynamic registration system that covers the entire growth cycle. This work is expected to provide robust technical support for precise phenotyping and elite cultivar selection in rubber tree breeding.

## 5. Conclusions

This paper introduced a robust and accurate registration method for seamlessly integrating ULS and HLS point clouds in complex rubber plantation environments. By semantically extracting stable wooden structures and proposing a novel WSK detection mechanism based on wood structure significance, the method effectively addresses the challenges posed by dense canopies and seasonal variations. Extensive evaluation across multiple sites and seasonal conditions demonstrates that the method achieves centimeter-level accuracy, proving its strong applicability in real-world phenotyping scenarios. The publicly released multi-season, multi-view dataset provides a valuable resource for future research. While the method shows significant promise, future work will focus on extending its applicability to continuous growth stages and integrating it into a comprehensive dynamic phenotyping system to further support high-throughput breeding and precision agriculture in rubber trees.

## Figures and Tables

**Table 1:** Technical specifications of ULS and HLS systems.

Platform	ULS	HLS
Instrument	CBI-Lite	LiGrip H120
Accuracy	5 cm@70 m	≤5 cm
Measurement rate	640,000 pts/s	320,000 pts/s
Wavelength	905 nm	905 nm
Range accuracy	120 m	120 m
Weight	1.28 kg	1.83 kg

**Table 2:** Summary of scanned sites.

Sites	Growth Stage	Plot Dimension (m^2^)	Tree Density (1/ha)	Avg. Height (m)	Canopy Density (%)	Collection Date	Canopy Complexity	Difficulty Level
Site #1	Mature	30 × 30	433	22	67	March	Simple	Low
Site #2	Mature	30 × 30	610	24	60	March	Simple	Low
Site #3	Mature	30 × 30	555	22	64	March	Simple	Low
Site #4	Mature	30 × 30	561	22	94	August	Complex	High
Site #5	Juvenile	30 × 30	1755	15	68	March	Simple	Low

**Table 3:** Performance evaluation of HLS-ULS registration in survey plot accuracy.

Survey Site	Angle Error (Degree)			Centroid Error (m)	RMSE (m)	Time (min)
	*θ_X_*	*θ_Y_*	*θ_Z_*			
Site #1	0.12	0.09	0.13	0.04	0.06	3.4
Site #2	0.08	0.16	0.15	0.09	0.10	4.35
Site #3	0.12	0.10	0.11	0.08	0.10	2.93
Site #4	0.34	0.45	0.47	0.13	0.14	3.48
Site #5	0.16	0.14	0.15	0.06	0.08	3.30
Average	0.16	0.19	0.20	0.08	0.09	3.49

**Table 4:** Performance Comparison of Keypoint Detectors in Registration.

Method	Keypoint Number		ANND (m)	Extraction Time (s)	RMSE (m)
	ULS	HLS			
WSK (ours)	18,027	19,242	0.37	7	0.06
Harris3D	18,526	19,116	0.38	3.6	0.13
ISS	17,076	18,697	0.34	3	0.07
SIFT	15,912	16,487	0.39	34.5	0.11

**Table 5:** Comparative Experiment.

Survey Site	Method	Angle Error (Degree)			Centroid Error (m)	RMSE (m)
		*θ_X_*	*θ_Y_*	*θ_Z_*		
Site #1	SAC-IA	3.02	2.70	2.02	0.25	0.40
	HRegNet	0.37	0.56	0.61	0.31	0.37
	Ours	0.12	0.09	0.13	0.04	0.06
Site #2	SAC-IA	0.86	0.94	1.00	0.24	0.34
	HRegNet	0.29	0.31	0.46	0.27	0.31
	Ours	0.08	0.16	0.15	0.09	0.10
Site #3	SAC-IA	10.81	11.71	4.02	7.59	11.27
	HRegNet	0.49	0.70	0.64	0.30	0.39
	Ours	0.12	0.10	0.11	0.08	0.10
Site #4	SAC-IA	3.78	3.98	1.70	0.97	1.61
	HRegNet	1.83	1.49	1.9	1.58	1.73
	Ours	0.34	0.45	0.47	0.13	0.14
Site #5	SAC-IA	9.72	10.25	3.06	6.19	5.58
	HRegNet	0.46	0.57	0.63	0.35	0.42
	Ours	0.16	0.14	0.15	0.06	0.08

**Table 6:** Performance in benchmarking public datasets.

Site ID	Tree Species	Angle Error (Degree)			Centroid Error (m)	RMSE (m)
		*θ_X_*	*θ_Y_*	*θ_Z_*		
S01	mixed	1.54	1.66	1.31	0.67	0.91
S02	willow	0.28	1.36	1.36	0.18	0.32
S03	poplar	0.50	0.84	0.87	0.12	0.17
S04	white birch	0.17	0.21	0.13	0.07	0.11
S05	mixed	0.32	0.22	0.30	0.05	0.12
S06	larch	0.11	0.36	0.38	0.10	0.19
S07	poplar	0.30	0.45	0.54	0.15	0.21
S08	*Pinus sylvestris*	0.34	0.34	0.44	0.11	0.28
S09	mixed	0.35	1.09	1.08	0.20	0.68
S10	eucalyptus	0.13	0.19	0.20	0.12	0.15
S11	fir	0.18	0.21	0.14	0.10	0.16
S12	fir	0.17	0.21	0.21	0.08	0.11
S13	fir	0.24	0.48	0.47	0.17	0.21
S14	*Pinus yunnanensis*	0.04	0.14	0.15	0.04	0.06
S15	eucalyptus	0.05	0.06	0.04	0.02	0.04
S16	eucalyptus	0.10	0.26	0.28	0.04	0.08
S17	*Pinus densata*	0.06	0.07	0.09	0.04	0.04

## Data Availability

The dataset is available at https://huggingface.co/datasets/TanJunxiang/ULS-HLS-Rubber/tree/main (accessed on 20 January 2026).
